# Plasma p-tau_217_ correlates strongly with cerebrospinal fluid Aβ_42_ and increases over a ten-year period in amyloid-positive, non-demented very old men

**DOI:** 10.1177/13872877251390387

**Published:** 2025-10-29

**Authors:** Elisabeth Hellquist, Shorena Janelidze, Bodil Weidung, Kristin Franzon, Vilmantas Giedraitis, Martin Ingelsson, Divya Bali, Vilma Velickaite, Henrik Zetterberg, Oskar Hansson, Lena Kilander

**Affiliations:** 1 Department of Public Health and Caring Sciences, Clinical Geriatrics, Uppsala University, Uppsala, Sweden; 2 Clinical Memory Research Unit, Lund University, Lund, Sweden; 3 Department of Public Health and Caring Sciences, Molecular Geriatrics, Rudbeck Laboratory, Uppsala University, Uppsala, Sweden; 4 Krembil Brain Institute, University Health Network, Toronto, Ontario, Canada; 5 Tanz Centre for Research in Neurodegenerative Diseases, Departments of Medicine and Laboratory Medicine & Pathobiology, University of Toronto, Toronto, Ontario, Canada; 6 Neuroradiology medical unit, Karolinska University Hospital, Stockholm, Sweden; 7 Department of Psychiatry and Neurochemistry, Institute of Neuroscience and Physiology, the Sahlgrenska Academy at the University of Gothenburg, Mölndal, Sweden; 8 Clinical Neurochemistry Laboratory, Sahlgrenska University Hospital, Mölndal, Sweden; 9 Department of Neurodegenerative Disease, UCL Institute of Neurology, Queen Square, London, UK; 10 UK Dementia Research Institute at UCL, London, UK; 11 Hong Kong Center for Neurodegenerative Diseases, InnoHK, Hong Kong, China; 12 Wisconsin Alzheimer’s Disease Research Center, University of Wisconsin School of Medicine and Public Health, University of Wisconsin-Madison, Madison, WI, USA

**Keywords:** Alzheimer's disease, biomarker, cross-sectional, longitudinal, plasma, p-tau217, population-based

## Abstract

**Background:**

Plasma phosphorylated tau_217_ (p-tau_217_) is a robust biomarker of Alzheimer's disease (AD) pathology. However, its full potential as a dynamic marker has still not been verified in very old persons, i.e., those with the highest incidence of AD.

**Objective:**

To examine the cross-sectional and longitudinal associations between plasma p-tau_217_ concentration and cerebrospinal fluid (CSF) AD biomarkers. Further, to investigate the performance of p-tau_217_ as a predictor of amyloid status in a cohort of very old men.

**Methods:**

CSF AD biomarkers were analyzed in thirty-five 89-year-old men. Amyloid-β (Aβ) positivity was defined according to CSF Aβ_42_ level. Plasma p-tau_217_ concentration was measured at the mean age of 82, 87, and 91. Incident dementia diagnoses in survivors were identified through medical records up to the age of 102.

**Results:**

Plasma p-tau_217_ strongly correlated with CSF Aβ_42_ concentration in Aβ-positive (n = 16, Spearman *ρ*: rho = −0.63, *p* = 0.009) but not in Aβ-negative (n = 19, rho = 0.111, *p* = 0.652) men and predicted Aβ status (area under the curve, AUC 0.91). Plasma p-tau_217_ increased over ten years in the Aβ-positive group, while it remained unchanged in the negative group (p = 0.018).

**Conclusions:**

Our findings indicate that plasma p-tau_217_ is a predictor of brain Aβ deposition also in very old individuals.

## Introduction

Dementia is one of the major causes of disability and dependency among older people worldwide.^1^ Alzheimer's disease (AD) is the most common dementia disorder, affecting millions of people and expecting to triple by 2050.^2^ The strongest risk factor for AD is high age. Cognitive deterioration progresses slowly and the stage of mild cognitive impairment (MCI) often lasts for several years, preceding loss of functions (i.e., dementia).^3^ The hallmarks of AD; brain accumulation of amyloid-β (Aβ) plaques and hyperphosphorylated tau (p-tau) neurofibrillary tangles, may be monitored by measuring the concentrations in cerebrospinal fluid (CSF) or by positron emission tomography (PET).^4,5^ Parallel to intracerebral aggregation, CSF Aβ_42_ declines over decades with the fastest rate in preclinical disease,^6^ remaining stationary already by the time cognitive symptoms appear.^4,^^7–9^ Later on, from the stage of MCI, CSF p-tau_181_ increases before reaching its plateau.^10^ Low concentrations of Aβ_42_ and high levels of p-tau_181_ in CSF strongly support a diagnosis of AD in patients with verified episodic memory decline. This holds true at the manifest dementia stage as well as in MCI.

PET is an expensive procedure and lumbar puncture may cause discomfort for the patient and it is not always possible to obtain CSF, due to anatomical reasons. Furthermore, these procedures are not available in a primary care setting where most elderly patients will present with cognitive symptoms. In primary care, the standard evaluation for diagnosing AD includes clinical examination, cognitive testing, and computed tomography (CT) scanning of the brain. Collection of blood is less invasive and less time-consuming than lumbar puncture (LP) or neuroimaging. Hence, establishing methods for analyzing AD plasma biomarkers is highly prioritized. Since it was developed, plasma p-tau has proven to be a robust biomarker of a multitude of features along the AD continuum.^11^

Of various p-tau isoforms (p-tau_181_, p-tau_217_, p-tau_231_), tau phosphorylated at threonine 217 (p-tau_217_) is the most sensitive marker of multiple changes associated with AD.^12^ Plasma p-tau_217_ has a high accuracy in discriminating Aβ-positive from Aβ-negative individuals; in identifying neuropathologically confirmed AD^13^; and in differentiating AD from other neurodegenerative disorders.^13,14^ It predicts decline in cognitive test performance, accelerated cerebral atrophy and cerebral glucose hypometabolism, as well as conversion from preclinical and prodromal stages to manifest AD dementia.^15–17^ Further, it shows the greatest magnitude of change over time compared to the other isoforms.^12,18^ In brief, plasma p-tau_217_ increases from the stage of PET amyloid-positivity and tau-negativity before cognitive impairment,^19^ and further on to AD MCI and mild AD dementia.^12^ These dynamics have mainly been described in cross-sectional analyses of memory research cohorts, including participants within the AD spectrum and with a majority of them being below 75 years of age. Only a few papers have reported serial measurements of plasma p-tau_217_^15,^^20–22^or p-tau_181_ in the same individuals, and with follow-up periods up to approximately five years.^23–27^ Before it can become a clinical routine in primary care diagnostics, the potential of plasma p-tau_217_ as a dynamic biomarker needs to be verified also in community-based populations.^28^ Not at least there is a need to achieve more knowledge about the dynamics in the oldest-old since several common conditions in high age may affect soluble p-tau. Cerebrovascular lesions, hypertension, diabetes and renal failure including other comorbidities^28^ can influence the permeability of the blood-brain-barrier and may also affect the metabolism of p-tau and the relationship with amyloid fibrils.^29^ Despite the numerous publications on plasma p-tau, to our knowledge, no previous study investigated its accuracy to predict amyloid status or the longitudinal changes in a very old cognitively unimpaired population.

In this study, we analyzed p-tau_217_ concentrations in plasma samples from an age- homogeneous cohort of non-demented elderly men who had also undergone LP. We included plasma sampled at three different time points over a ten-year period from the mean age of 82 to study the trajectory of plasma p-tau_217_, its correlations with standard CSF AD biomarkers and performance in predicting CSF amyloid status. Our secondary aim was to investigate if plasma p-tau_217_ correlated with performance in the Mini-Mental State Examination (MMSE) and with the degrees of global cortical atrophy (GCA) and medial temporal lobe atrophy (MTA) according to CT. Finally, we explored if plasma p-tau_217_ was associated with incident dementia.

## Methods

The study individuals were participants in the Uppsala Longitudinal Study of Adult Men (ULSAM), a prospective cohort that started in 1970, described at ULSAM - Uppsala University (uu.se). All 50-year-old men born in 1920–1924 living in Uppsala, Sweden were invited to a health survey, initially focusing at identifying risk factors for cardiovascular disease. Eighty-two percent (n = 2322) participated in the first investigation at the age of 50. The participants were thereafter invited to examinations at ages approximately 60, 70, 77, 82 (U-5), 87 (U-6), and 91 (U-7) years. All participants were of European ancestry. Previous research on this cohort has demonstrated a strong concordance between educational level and socioeconomic status.^30^ An experienced research nurse administered the MMSE^31^ and collected plasma samples at U-5, U-6, and U-7.

**Figure 1.**
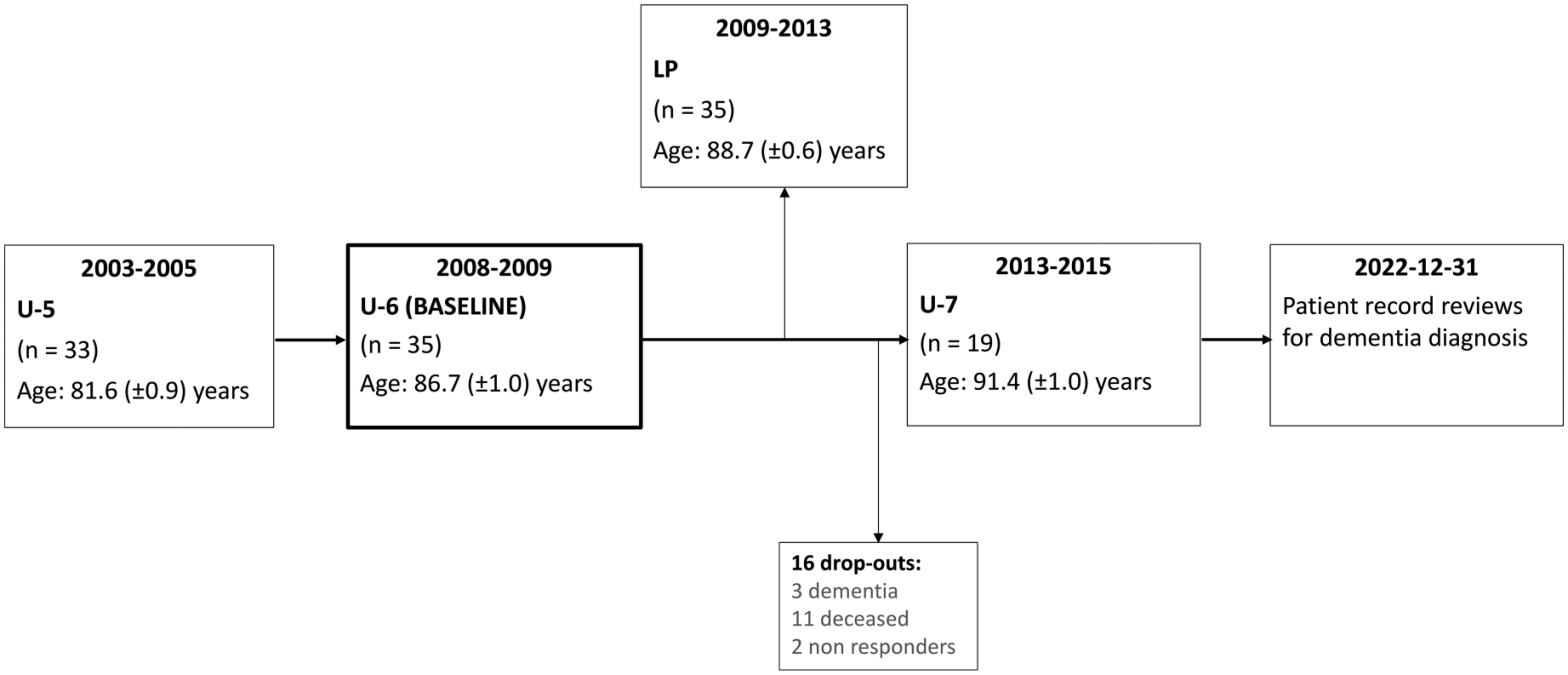
Flowchart of the participants in the CSF group. LP: lumbar puncture.

Baseline for this study was U-6, which took place in 2008–2009 when the participants were aged 84–88 years, n = 352, as previously described.^32,33^ For this study we only included men without diagnosed dementia at baseline. One hundred and sixty participants were not on warfarin treatment and fit enough to be considered eligible for LP. They were invited to a sub study with CT of the brain and LP approximately two years later. Fifty-seven individuals agreed to participate and underwent CT and 52 of the 57 subjects accepted to undergo LP. Due to anatomical reasons, LP could only be completed in 35 subjects and these constitute our primary study group (Figure 1 and Table 1).

**Table 1.** Characteristics of the participants.

	CSF group		CT group		All participants	
	N	Statistics	N	Statistics	N	Statistics
Age at U-6 (mean, SD), y	35	86.7 (±1.0)	57	86.8 (±1.0)	352	86.7 (±1.1)
Age at LP (mean, SD), y	35	88.7 (±0.6)				
Time between plasma sample and LP (mean, SD), y						
U-5	33	-7.1 (±0.7)				
U-6	35	-2.0 (±0.8)				
U-7	19	+2.9 (±0.8)				
*APOE* ε4 allele carrier, n (%)	34	3 (8.8)	56	4 (7.1)	327	84 (25.7)
Educational level, n (%)	35		57		352	
Low		14 (40)		28 (49.1)		182 (51.7)
Medium		14 (40)		18 (31.6)		107 (30.4)
High		7 (20)		11 (19.3)		63 (17.9)
MMSE score, p (median, IQR)						
U-5	33	29 (28–30)	54	29 (28–30)	274	28 (27–29)
U-6	35	29 (27–30)	57	28 (27–29)	351	28 (26–29)
U-7	18	26 (25–28)	31	26 (25–28)	105	27 (25–28)
Charlson score at U-6, n (%)	35		57		352	
0		15 (43)		26 (46)		122 (35)
1		8 (23)		13 (23)		92 (26)
≥ 2		12 (34)		18 (31)		138 (39)
Plasma p-tau_217_ (median, IQR), pg/ml						
U-5	33	0.33 (0.24–0.39)	54	0.32 (0.24–0.38)	510	0.32 (0.25–0.39)
U-6	35	0.36 (0.27–0.50)	57	0.36 (0.27–0.53)	352	0.36 (0.27–0.53)
U-7	19	0.41 (0.28–0.54)	32	0.45 (0.29–0.64)	111	0.44 (0.31–0.62)
CSF- Aβ_42_ (median, IQR), pg/ml	35	703 (482–927)				
Aβ-positive (<620 pg/ml), n (%)		16 (46)				
CSF p-tau_181_ (median, IQR), pg/ml	35	61 (48–79)				
CSF t-tau (median, IQR), pg/ml	35	415 (344–617)				
CT MTA score, n (%)	34		57			
1–2		23 (68)		38 (67)		
3–4		11 (32)		19 (33)		
CT GCA score, n (%)	34		57			
1		16 (47)		22 (38.5)		
2		17 (50)		34 (59.5)		
3		1 (3)		1 (2)		
Incident dementia n (%)	35	9 (26)	57	15 (26)	352	115 (33)

CSF: cerebrospinal fluid; CT: computer tomography; LP: lumbar puncture; *APOE* ε4: Apolipoprotein ε4; MMSE: Mini-Mental State Examination; MTA: medial temporal lobe atrophy; GCA: global cortical atrophy

Plasma p-tau_217_ concentrations were measured in archived samples from all participants in U-5 (n = 510), U-6 (n = 352), and U-7 (n = 125). In the CSF group, in samples from U-5 (n = 33), U-6 (n = 35), and U-7 (n = 19); and in the CT group in samples from U-6 (n = 57). The plasma samples were stored at −70°C until the analyses. The concentrations were measured at the Memory Research Unit, Lund University, using an immunoassay on a Meso Scale Discovery platform developed by Lilly Research Laboratories. Briefly, biotinylated-IBA493 was used as a capture antibody and SULFO-TAG-4G10-E2 (anti-Tau) as the detector and samples were diluted 1:2. The assay was calibrated with a synthetic p-tau_217_ peptide. All samples were analyzed in duplicates and by staff blinded to the clinical and imaging data. 0.6% (3/510) of p-tau_217_ values were below the lower detection limit of the assay (0.15 pg/ml).

The CT scans were performed at the Uppsala University Hospital, as previously described.^34^ An 8-slice scanner (General Electric, Boston, MA) or a 64-slice scanner (Siemens Healthengineers, Erlangen, Germany) was used to acquire the images. The CT images were reformatted to axial, sagittal and coronal planes, with a slice thickness of 4 mm. All images were independently reviewed by two neuroradiologists, both blinded to cognitive status and CSF findings. The degree of the frontal atrophy was graded according to the Pasquier scale for GCA^35,36^ and the degree of MTA was graded using the Scheltens scale.^37^ These scales are graded from 0 (no atrophy), to either 3 (Pasquier scale) or 4 (Scheltens scale), corresponding to the most severe degree of atrophy. In cases of disagreement between the neuroradiologists a consensus evaluation was made to reach the final scoring results. The CT scans were performed 1.7 (±0.9) years after U-6.

Lumbar puncture was performed by one investigator with the patient in a lying position. Twelve ml of CSF were collected in polypropylene tubes and samples with clear visual blood contamination were excluded. The samples were centrifuged and aliquoted in 1.5 ml polypropylene tubes, followed by storage at −70°C until the analyses. The lumbar punctures were performed 0.3 (±0.4) years after the CT scans (Figure 1).

The concentrations of Aβ_42_, t-tau, and p-tau_181_ in CSF were analyzed at the Clinical Neurochemistry Laboratory, University of Gothenburg, Mölndal, Sweden. The analyses were performed by board-certified laboratory technicians, who were blinded to clinical data, using sandwich ELISA (INNOTEST, Fujirebio, Ghent, Belgium) and standardized procedures accredited by the Swedish Board of Accreditation and Conformity Assessment. Aβ positivity was defined as a CSF Aβ_42_ level ≤620 pg/ml as defined by the laboratory's established clinical reference ranges.

Dementia diagnoses were assigned by two independent, experienced geriatricians, without information of the CSF biomarkers.^38^ Follow-up diagnostics were made using all data available in medical records from Uppsala University Hospital, primary care, and nursing homes in Uppsala County until December 31, 2022, i.e., up to age of 102 in survivors. In case of disagreement, a third experienced geriatrician reviewed the case, and the diagnosis was determined by a majority decision.

Dementia was defined according to the criteria from DSM-IV,^39^ in brief a persisting cognitive deterioration severe enough to interfere with activities of daily life, with other somatic or psychiatric disorders ruled out as explanations. AD was diagnosed according to the National Institute of Neurological and Communicative Diseases and Stroke and the Alzheimer's disease and Related Disorders Association (NINCDS-ADRDA) criteria,^40^ including findings from the CT scans. Cases of dementia without neuroimaging and without sufficient clinical details in the medical records to set a specific dementia subtype diagnosis were classified as unspecified dementia.

Genotyping for apolipoprotein E (*APOE*) by minisequencing^41^ was performed in 327 of the 352 participants at U-6. The National Patient Registry provided information on in-patient care before baseline at U-6, and this information was used to calculate the Charlson Comorbidity Index.^42,43^ The study was approved by the local ethical committee (Reference number: Dnr 02-605, Dnr 2007/338 and Dnr 2013/350), and all participants gave their written informed consent.

### Statistics

Distribution or normality of variables were examined by visual assessment of histogram and standard error of skewness.

Spearman's correlation was used to calculate the correlations between plasma p-tau_217_ and CSF biomarkers, CT findings and MMSE, respectively. Two-tailed values of p < 0.05 were considered statistically significant. A mixed model was used to calculate the differences in plasma p-tau_217_ trajectory over time with respect to Aβ-status. Due to the small group sizes, it was not possible to include random intercepts in the mixed model. To examine the discriminative performance of plasma p-tau_217_ for predicting Aβ status we used the area under the receiver operating characteristic curve (ROC).

The Mann-Whitney U test was used to compare levels of plasma p-tau_217_ and CSF Aβ_42_ between participants with and without incident dementia, as the variables were not normally distributed.

To compare the age between non-participants (the ones with plasma samples who were not included in the CT or CSF group (n = 295)) and the participants T-test was used. Non-parametric analyses, including Mann-Whitney U and Chi-Square test, were used to compare education level, Charlson score and *APOE* ε4-carriership.

All statistical analysis were performed using IBM SPSS Statistics (ver.28.0.1.0 for Windows; IBM Corporation, Armonk, NY, USA)

## Results

Characteristics of the participants in the study are shown in Table 1. At baseline (U-6), the mean (SD) age of the 35 participants in the CSF group was 86.7 (±1.0) years and 88.7 (±0.6) years at the time for LP. We found no significant age differences at plasma samplings, educational level or baseline Charlson score between the CSF group and all U-6 participants.

Only nine percent (3/34) of the CSF group were *APOE* ε4 allele carriers, which was significantly lower in comparison to 26% carriers in the total U-6 sample (*p* < 0.05). Sixteen participants were defined as Aβ-positive and 19 as Aβ-negative. The MMSE score distribution at baseline were within the normal range (median = 29 p, IQR = 27–30 p). Nineteen men took part in U-7; of the remaining sixteen, eleven were deceased, three were diagnosed with dementia and two did not respond.

There were no significant differences in plasma p-tau_217_ concentration in the CSF group compared with the whole sample at all three examinations. Plasma p-tau_217_ concentrations were in strong agreement across the different sampling ages (U-5 versus U-6: Spearman *ρ*: rho = 0.81, *p* < 0.001; U-6 versus U-7: Spearman *ρ*: rho = 0.82, *p* < 0.001).

**Figure 2.**
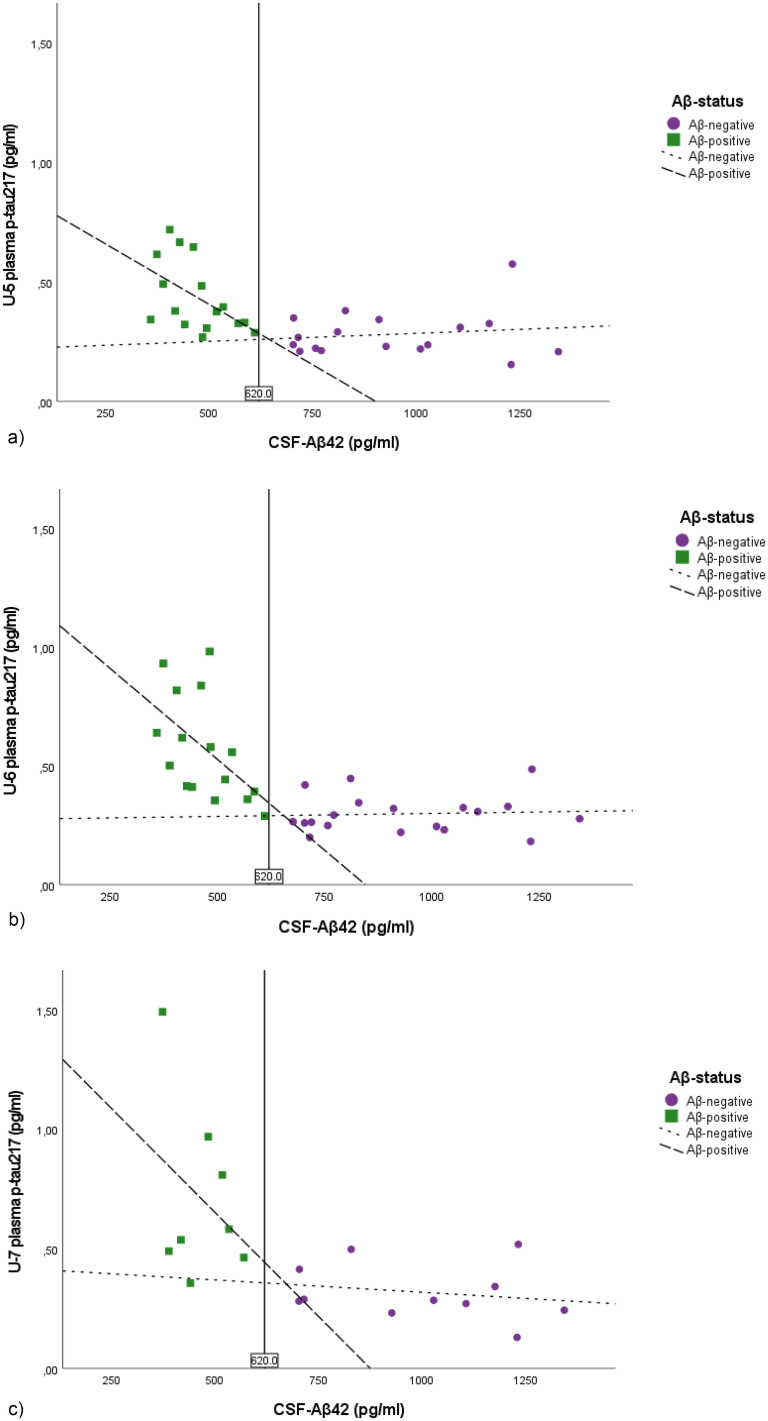
Scatter plots of CSF Aβ_42_ and plasma p-tau_217_ at the different examinations. Figure 2(a)**.** Scatter plots of CSF Aβ_42_ and plasma p-tau_217_ seven years before CSF sampling (U-5). Figure 2(b)**.** Scatter plots of CSF Aβ_42_ and plasma p-tau_217_ two years before CSF sampling (U-6, baseline). Figure 2(c). Scatter plots of CSF Aβ_42_ and plasma p-tau_217_ three years after CSF sampling (U-7).

Figure 2a-c shows the strong inverse correlations between CSF Aβ_42_ and plasma p-tau_217_ concentrations at U-5, U-6, and U-7 (Spearman *ρ*: rho = −0.59, *p* < 0.001, rho = −0.69, *p* < 0.001 and rho = −0.67, *p* = 0.002). In the Aβ-positive group, cross-sectionally, CSF Aβ_42_ inversely correlated with plasma p-tau_217_ at U-5 (n = 16, Spearman ρ: rho −0.55 p = 0.026), U-6 (n = 16, Spearman ρ: rho −0.63 p = 0.009) but not at U-7 (n = 8, Spearman ρ: rho −0.24 p = 0.57). In the Aβ-negative group there was no significant correlation between plasma p-tau_217_ and CSF Aβ_42_ at any of the examinations U-5 (n = 17, Spearman ρ: rho −0.0,76 p = 0.772), U-6 (n = 19, Spearman ρ: rho 0.111 p = 0.652) and U-7 (n = 11, Spearman ρ: rho −0.20 p = 0.555).

**Figure 3.**
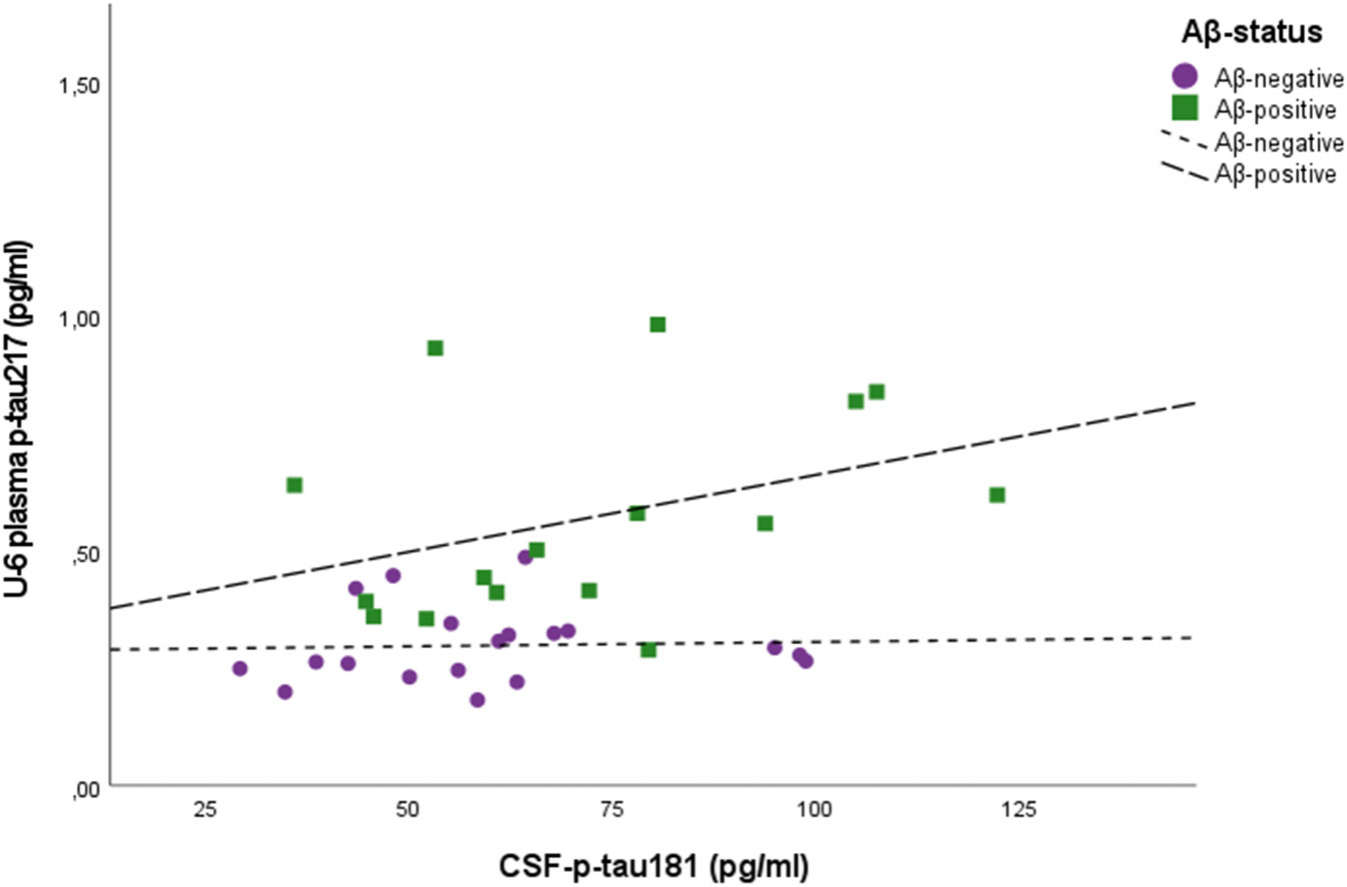
Scatter plots of CSF p-tau_181_ and plasma p-tau_217_ two years before CSF sampling (U-6, baseline).

Figure 3 shows the correlation of plasma p-tau_217_ at baseline with CSF p-tau_181_ concentrations. The correlations of plasma p-tau_217_ with CSF p-tau_181_ concentration were of borderline significance at U-5 and U-6 (Spearman *ρ*: rho = 0.33, *p* = 0.058; rho = 0.33, *p* = 0.051) but did not exist at U-7 (Spearman *ρ*: rho = 0.20, *p* = 0.412), (Supplemental Figure 1a-(c)). The corresponding figures for Aβ-positive subjects were: U-5; n = 16, (Spearman *ρ*: rho = 0.39 *p* = 0.131), U-6; n = 16 (Spearman *ρ*: rho = 0.41, *p* = 0.116) and U-7; n = 8 (Spearman *ρ*: rho = 0.05, *p* = 0.911).

Plasma p-tau_217_ concentration did not correlate with CSF total tau; at U-5, U-6, and U-7, respectively (Spearman *ρ*: rho = 0.24, *p* = 0.178; rho = 0.29, *p* = 0.091, and rho = 0.225, *p* = 0.335).

**Figure 4.**
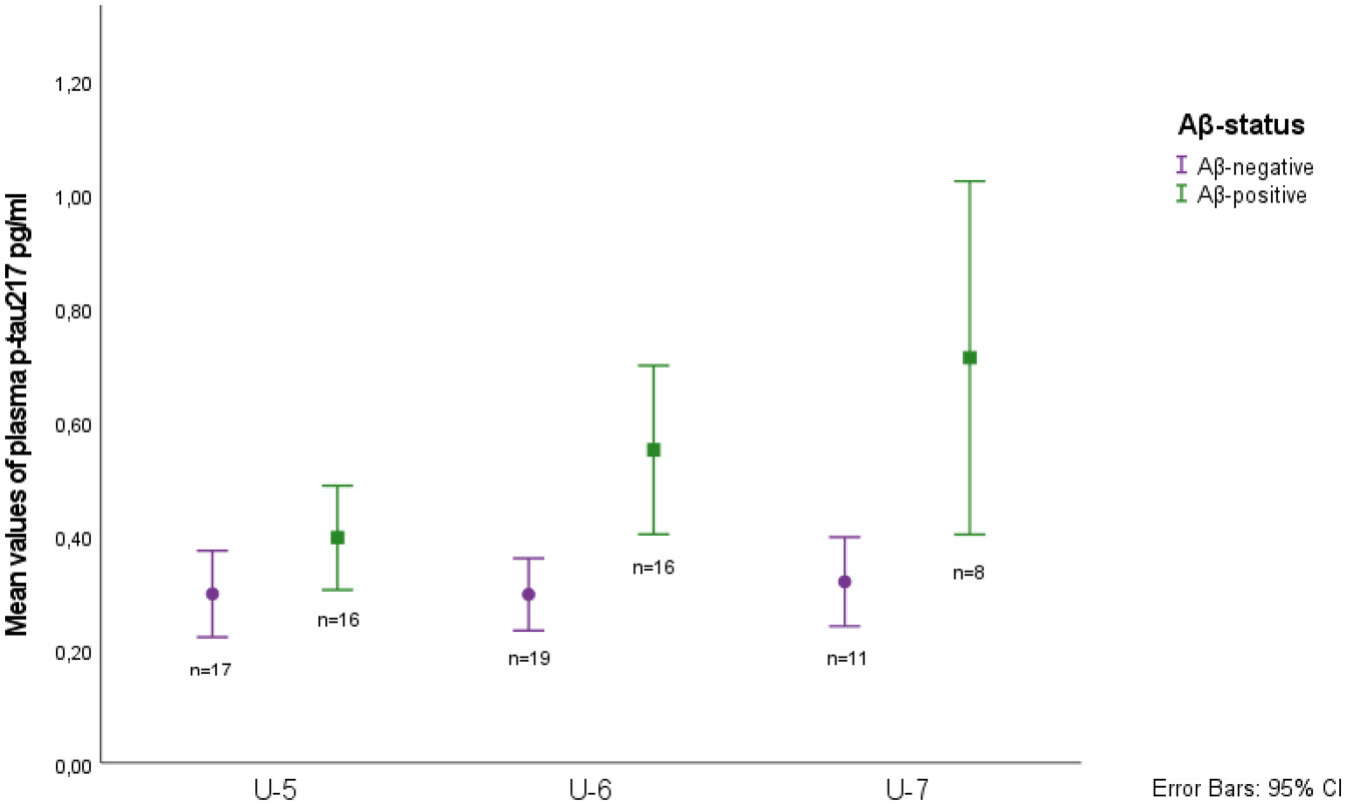
Box plots of mean values of plasma p-tau_217_ concentrations according to Aβ-status at the three different investigations.

Table 2 shows that median plasma p-tau_217_ concentrations differed cross sectionally by CSF Aβ status at all three examinations. Figure 4 illustrates the longitudinal trajectory of plasma p-tau_217_, which differed significantly by CSF Aβ status (*p* = 0.018). There was an increasing trend in the Aβ-positive group (B = 0.137, 95% CI = 0.039–0.235, *p* = 0.007), whereas levels remained unchanged over time in the Aβ-negative group (*p* = 0.302).

**Table 2.** Concentrations of plasma p-tau_217_ at the three examinations considering Aβ status.

	Aβ-positive		Aβ-negative		Statistics
	n	Plasma p-tau_217_ (median, IQR), pg/ml	n	Plasma p-tau_217_ (median, IQR), pg/ml	p, (Mann-Whitney U test)
U-5	16	0.38 (0.32–0.58)	17	0.24 (0.22–0.33)	p < 0.001
U-6	16	0.53 (0.40–0.77)	19	0.28 (0.25–0.33)	p < 0.001
U-7	8	0.56 (0.47–0.93)	11	0.29 (0.24–0.41)	p = 0.002

**Figure 5.**
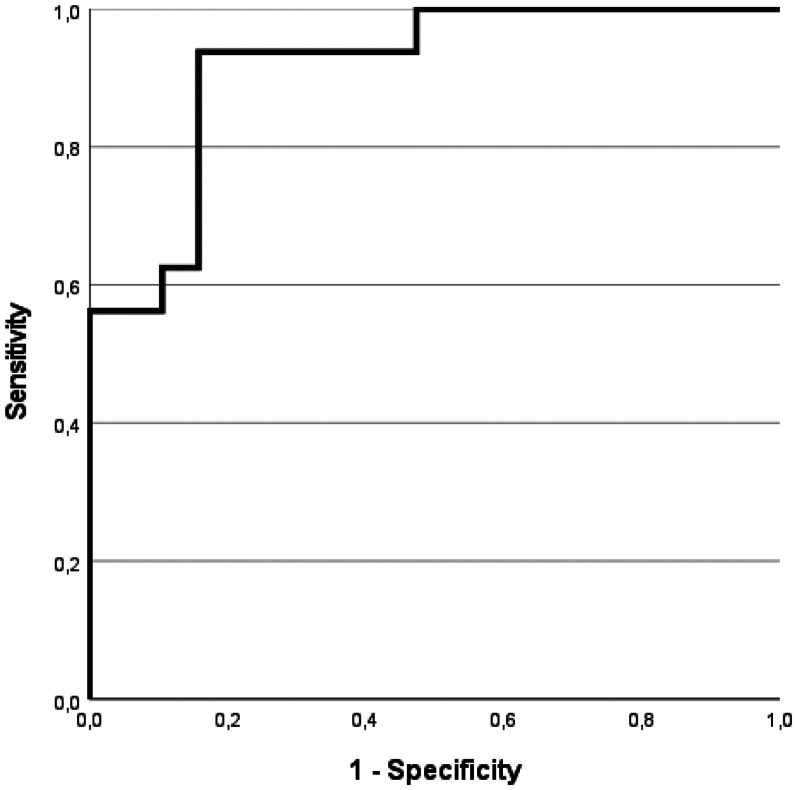
Receiver operating characteristics (ROC) curve for plasma p-tau_217_ in distinguishing Aβ-positive subjects from Aβ-negative subjects.

As shown in Figure 5, plasma p-tau_217_ demonstrated strong discriminatory ability between Aβ-positive and Aβ-negative individuals (AUC = 0.91, 95% CI: 0.82–1.0, *p* < 0.001).

Among the 57 individuals that underwent CT scan, 33% had an MTA score of 3–4 and 62% a GCA score of 2–3. There were no significant correlations between plasma p-tau_217_ at baseline and any of the neuroradiological markers, for MTA (Spearman *ρ*: rho = −0.01, *p* = 0.937) and for GCA (Spearman *ρ*: rho = 0,109, *p* = 0.421).

Further, there was no significant correlation between plasma p-tau_217_ and MMSE scores at baseline. Spearman *ρ*: rho = −0.09, *p* = 0.619 for the 35 individuals in the CSF group.

Incident dementia in the total U-6 population (n = 352) was 33%. In the CSF group, nine men (26%; four Aβ-positive and five Aβ-negative subjects) developed dementia during follow-up to December 2022, or to the date of death. Six men had received a clinical diagnosis of AD (without knowledge of the CSF data) and in three cases no specific dementia diagnosis could be set. Although the confidence intervals were overlapping, non-significantly higher plasma p-tau_217_ levels were seen at baseline in men with incident dementia (median 0.42 pg/ml, IQR [0.29–0.79] versus 0.34 pg/ml, [0.26–0.46], *p* = 0.21), whereas there was a trend for lower CSF Aβ_42_ levels (median 482 pg/ml, IQR [396–807] versus 717 pg/ml, [512–1014], *p* = 0.12).

## Discussion

In this community-based cohort of 35 very old men, free from dementia at baseline, we found a close correlation between levels of plasma p-tau_217_ and CSF Aβ_42_ in amyloid-positive subjects. Importantly, plasma p-tau_217_ proved to be a dynamic AD biomarker with markedly increasing concentrations in the Aβ-positive group over a ten-year period, while it was unchanged in Aβ-negative participants. These results support that elevated plasma p-tau_217_ primarily reflects the intracerebral aggregation of Aβ-fibrils in the preclinical AD stage in very high age, as has been shown previously among younger age groups.

In our cohort, the correlation between plasma p-tau_217_ and CSF Aβ_42_ was present already at U-5, five years prior to baseline, when plasma p-tau_217_ not yet differed between Aβ-positive and Aβ-negative subjects. Plasma p-tau_217_ shows a steep increase after Aβ biomarkers have become positive.^44^ An association between plasma p-tau and amyloid accumulation according to PET, already in the asymptomatic stage, has been demonstrated in large memory research cohorts mainly of older individuals, although younger than in our cohort.^10,12,15,45^ Fewer studies have reported the associations between plasma p-tau and CSF amyloid markers. In BioFINDER-2, where the majority were cognitively healthy or mildly impaired and mean age approximately seventy, plasma p-tau_217_ had a moderate correlation with the CSF Aβ_42/40_ ratio.^45^ Our results align with previous studies showing plasma p-tau_217_'s performance to detect pathological CSF Aβ_42/40_ ratio in individuals with MCI and mean age approximately 72 years (AUC 0.858–0.947).^16^ In the BioFINDER-1 cohort AUC was 0.783 for cognitively unimpaired and 0.879 for MCI subjects.^15^

The underlying mechanisms behind pathological soluble tau metabolism are incompletely understood. Increasing plasma p-tau seems to represent physiological reactions to Aβ aggregation at early stages.^12,29^ We found no correlation between plasma p-tau and CSF Aβ in men with negative amyloid status, indicating that high age *per se* does not uncouple the connection between intracerebral Aβ and peripheral soluble p-tau.

In previous studies,^12^ plasma p-tau_217_ concentrations showed weaker correlation with CSF p-tau_181_ than with CSF Aβ_42_. It was of borderline significance in our cohort probably due to lack of power. Other studies have reported a strong concordance between plasma p-tau and CSF p-tau in amyloid positive individuals across the AD spectrum, with a closer relationship for plasma p-tau_217_ than plasma p-tau_181_.^16,29,45^ Earlier findings suggest a close correlation between plasma p-tau_217_ and CSF p-tau_217_ already in amyloid positive cognitively unimpaired subjects as well as in MCI, and prior to insoluble tau aggregates were detected by PET.^19^ In a recent narrative review of 33 heterogeneous studies, it was concluded that the strongest association between plasma and CSF p-tau exists in the MCI stage, being less pronounced in earlier stages and disappearing after plasma p-tau reaches its plateau.^29^ With a few exceptions, most studies showed moderate to strong correlations (R > 0.5) between plasma and CSF values,^14,16,18,^^45–47^ i.e., higher than in our cohort. The participants in our study were cognitively unimpaired at baseline and this may have contributed to the weak correlation between p-tau in plasma and CSF. We found no correlation between plasma p-tau_217_ and CSF total tau, which is consistent with earlier studies,^18^ and also not unexpected since total tau is a non-specific marker of neurodegeneration.

In the Aβ-positive men in our cohort, plasma p-tau_217_ steadily increased over a ten-year period. We have not identified any other study aiming to describe the trajectory of plasma p-tau in the same individuals over such an extended time period. A few previous studies have analyzed repeated measurements of plasma p-tau_217_ over 4–6 years, together with markers of AD progression. In the BioFINDER study, mean age approximately 70 years, preclinical and prodromal AD had accelerated plasma p-tau_217_ while it did not change over time in Aβ-negative participants, nor in MCI patients who did not convert to AD.^21^ In the Wisconsin Register for Alzheimer's Prevention cohort (WRAP), mean age 63 years, plasma p-tau_217_ demonstrated marked amyloid-dependent changes in both preclinical and prodromal AD, and was associated with declining cognition.^15^ In the same cohort, p-tau_217_ increased modestly with age in amyloid negative subjects.^22^ In a recent study, plasma p-tau_217_ was analyzed with a novel commercial immunoassay repeatedly over five years in the WRAP cohort. Plasma p-tau_217_ increased annually only in Aβ-positive subjects, and most markedly in those who also had a positive tau PET scan.^20^

We found no correlation between plasma p-tau_217_ and MTA, or with GCA in this small cohort. Such structural changes are commonly seen also in cognitively healthy subjects older than 80 years^48,49^ and CT scans (as used in the present study) are less sensitive for detecting brain atrophy than MRI imaging. Similarly, we did not find any correlation between plasma p-tau_217_ levels and the results on the MMSE in this small cohort, possibly due to the ceiling effects.

While the diagnostic and prognostic, i.e., conversion to AD dementia, performance of p-tau_217_ has been convincingly shown in large research cohorts^13,^^15–17^ our findings did not show significant associations with incident dementia, which may reflect the restricted sample size of our cohort. Dementia diagnoses were based on medical records, which may have led to underestimation of cases. However, Sweden's universal healthcare and our long follow-up to death likely reduced underreporting.

Further, five of the nine men that later were diagnosed with dementia were Aβ-negatives, reflecting that non-AD neuropathology such as cerebrovascular lesions, hippocampal sclerosis, TDP-43 accumulation and Lewy bodies are common in ninety-year-olds with dementia.^50,51^

Concerning representability, the number of *APOE* ε4 carriers was markedly lower in our sample than in the whole cohort. Although the Charlson Comorbidity Index scores did not differ between participants and non-participants, individuals eligible for LP (which were selected for the present study) were likely healthier and with less cognitive impairment compared to the others. The relatively low proportion of *APOE* ε4 carriers among participants may reflect this selection bias. However, the proportion of participants with positive amyloid status (46%) was very similar to other cohorts in the same age groups. One CSF study of cognitively unimpaired subjects reported that approximately 47% in 85-89-year-olds,^52^ and around 40% of 80–89 year-olds without dementia were positive according to amyloid PET.^53^ The present cohort included only men, which restricts the generalizability of the findings. Most studies that compared p-tau levels in serum or plasma did not find any differences between men and women, although some studies reported higher plasma p-tau_181_ or p-tau_217_ levels in men than in women, while elevated levels have been associated with greater cognitive decline in women.^54^

As the population ages and more people reach a very advanced age, the incidence of dementia increases.^55^ With current diagnostic tools in primary care where most elderly individuals with cognitive complaints initially present, the diagnostic accuracy for AD is around 60%.^56^ This results in suboptimal treatment and care. A recent study demonstrated that a diagnostic algorithm utilizing plasma p-tau_217_ accurately diagnosed AD in approximately 80% of patients with MCI.^57^ A blood biomarker to detect AD can be used in clinical settings for biological staging of the disease, and monitoring disease progression. This improved diagnostic accuracy can facilitate the initiation of widely accessible treatments, such as cholinesterase inhibitors. However, a pathological plasma p-tau value in older individuals with cognitive impairment should be interpreted with caution: it likely indicates Aβ plaque pathology but may not necessarily be causal, as previously mentioned. Not all individuals with amyloid pathology develop cognitive impairment. By the age of 95, individuals who die with and without dementia exhibit a similar burden of neuropathological changes.^58^ Hence, a positive plasma p-tau should not be used as a standalone diagnostic test for AD but must be interpreted within a clinical context.^59^ A diagnosis must benefit the patient and biomarker analysis should only be conducted when there is a reasonable suspicion of AD. Based on our findings, it is conceivable that repeated measurements in individuals with perceived cognitive complaints could guide clinical decisions. Correspondingly, normal values, especially in serial measurements, may inform the patient of a low risk of AD.

### Strengths and limitations

This population-based cohort was homogeneous with respect to age and cognitive level, and with amyloid status representative for this age group. To our knowledge this cohort is unique with both CSF samples and serial plasma p-tau_217_ concentration measurements beyond the age of ninety years and over a decade. There was a low rate of non-response among men who were still alive at the last sampling. Compared to PET, CSF biomarkers are continuous and change earlier in the AD continuum. Despite the small sample size, we observed robust correlations between plasma p-tau_217_ and CSF Aβ_42_. There are several limitations to this study. The small and selective sample, and the difficulties recruiting very old individuals for lumbar puncture, introduce potential selection bias and limit statistical power and generalizability. The limited number of participants may have precluded us from detecting an association to incident dementia. As previously discussed, dementia cases may have been underestimated, potentially reducing statistical power and precision.

CSF was collected approximately two years after baseline plasma samplings, hence, the number of Aβ-positives may be slightly overestimated. The CSF Aβ_42_, t-tau and p-tau_181_ ELISA assays used (INNOTEST) are subject to analytical variability and manual processing, which may affect sensitivity. However, all CSF analyses followed accredited, standardized procedures.

### Conclusion

In this study of very old men without diagnosed dementia, plasma p-tau_217_ was a reliable predictor of amyloid status, similar to younger age groups. Moreover, our data show that plasma p-tau_217_ is stable over a time period of at least ten years in Aβ-negative subjects even after the age of ninety. These findings indicate usefulness of this biomarker in clinical practice by aiding in disease progression monitoring and may offer the same information as CSF. This is highly relevant when deciding on pharmacological treatment, both currently and in light of future changes in the therapeutic landscape. The full potential of plasma p-tau_217_ as a prognostic biomarker of cognitive deterioration in very old persons needs to be further explored in larger cohorts including both genders.

## Supplemental Material

sj-docx-1-alz-10.1177_13872877251390387 - Supplemental material for Plasma p-tau_217_ correlates strongly with cerebrospinal fluid Aβ_42_ and increases over a ten-year period in amyloid-positive, non-demented very old men Supplemental material, sj-docx-1-alz-10.1177_13872877251390387 for Plasma p-tau_217_ correlates strongly with cerebrospinal fluid Aβ_42_ and increases over a ten-year period in amyloid-positive, non-demented very old men by Elisabeth Hellquist, Shorena Janelidze, Bodil Weidung, Kristin Franzon, Vilmantas Giedraitis, Martin Ingelsson, Divya Bali, Vilma Velickaite, Henrik Zetterberg, Oskar Hansson and Lena Kilander in Journal of Alzheimer's Disease

## References

[1] WHO . Dementia: Key facts. https://www.who.int/news-room/fact-sheets/detail/dementia, (accessed 2025-02-06).

[2] Nichols E Steinmetz JD Vollset SE , et al. Estimation of the global prevalence of dementia in 2019 and forecasted prevalence in 2050: an analysis for the Global Burden of Disease Study 2019. Lancet Public Health 2022; 7: E105–E125. 10.1016/S2468-2667(21)00249-8 PMC8810394 34998485

[3] Busche MA Hyman BT . Synergy between amyloid-beta and tau in Alzheimer's disease. Nat Neurosci 2020; 23: 1183–1193. 32778792 10.1038/s41593-020-0687-6 PMC11831977

[4] Blennow K Mattsson N Scholl M , et al. Amyloid biomarkers in Alzheimer's disease. Trends Pharmacol Sci 2015; 36: 297–309. 25840462 10.1016/j.tips.2015.03.002

[5] Chetelat G Arbizu J Barthel H , et al. Amyloid-PET and (18)F-FDG-PET in the diagnostic investigation of Alzheimer's disease and other dementias. Lancet Neurol 2020; 19: 951–962. 33098804 10.1016/S1474-4422(20)30314-8

[6] Lo RY Hubbard AE Shaw LM , et al. Longitudinal change of biomarkers in cognitive decline. Arch Neurol 2011; 68: 1257–1266. 21670386 10.1001/archneurol.2011.123 PMC5604752

[7] Jack Jr CR Knopman DS Jagust WJ , et al. Hypothetical model of dynamic biomarkers of the Alzheimer's pathological cascade. Lancet Neurol 2010; 9: 119–128. 20083042 10.1016/S1474-4422(09)70299-6 PMC2819840

[8] Rosén C Hansson O Blennow K , et al. Fluid biomarkers in Alzheimer's disease - current concepts. Mol Neurodegener 2013; 8: 20. 23800368 10.1186/1750-1326-8-20 PMC3691925

[9] Palmqvist S Zetterberg H Blennow K , et al. Accuracy of brain amyloid detection in clinical practice using cerebrospinal fluid beta-amyloid 42: a cross-validation study against amyloid positron emission tomography. JAMA Neurol 2014; 71: 1282–1289. 25155658 10.1001/jamaneurol.2014.1358

[10] Ossenkoppele R van der Kant R Hansson O . Tau biomarkers in Alzheimer's disease: towards implementation in clinical practice and trials. Lancet Neurol 2022; 21: 726–734. 35643092 10.1016/S1474-4422(22)00168-5

[11] Hansson O Blennow K Zetterberg H , et al. Blood biomarkers for Alzheimer's disease in clinical practice and trials. Nat Aging 2023; 3: 506–519. 37202517 10.1038/s43587-023-00403-3 PMC10979350

[12] Telser J Grossmann K Wohlwend N , et al. Phosphorylated tau in Alzheimer's disease. Adv Clin Chem 2023; 116: 31–111. 37852722 10.1016/bs.acc.2023.05.001

[13] Palmqvist S Janelidze S Quiroz YT , et al. Discriminative accuracy of plasma phospho-tau217 for Alzheimer disease vs other neurodegenerative disorders. JAMA 2020; 324: 772–781. 32722745 10.1001/jama.2020.12134 PMC7388060

[14] Thijssen EH La Joie R Strom A , et al. Plasma phosphorylated tau 217 and phosphorylated tau 181 as biomarkers in Alzheimer's disease and frontotemporal lobar degeneration: a retrospective diagnostic performance study. Lancet Neurol 2021; 20: 739–752. 34418401 10.1016/S1474-4422(21)00214-3 PMC8711249

[15] Ashton NJ Janelidze S Mattsson-Carlgren N , et al. Differential roles of Aβ42/40, p-tau231 and p-tau217 for Alzheimer's trial selection and disease monitoring. Nat Med 2022; 28: 2555–2562. 36456833 10.1038/s41591-022-02074-w PMC9800279

[16] Janelidze S Bali D Ashton NJ , et al. Head-to-head comparison of 10 plasma phospho-tau assays in prodromal Alzheimer's disease. Brain 2023; 146: 1592–1601. 36087307 10.1093/brain/awac333 PMC10115176

[17] Mattsson-Carlgren N Salvado G Ashton NJ , et al. Prediction of longitudinal cognitive decline in preclinical Alzheimer disease using plasma biomarkers. JAMA Neurol 2023; 80: 360–369. 36745413 10.1001/jamaneurol.2022.5272 PMC10087054

[18] Barthélemy NR Horie K Sato C , et al. Blood plasma phosphorylated-tau isoforms track CNS change in Alzheimer’s disease. J Exp Med 2020; 217: e20200861. 10.1084/jem.20200861 PMC7596823 32725127

[19] Janelidze S Berron D Smith R , et al. Associations of plasma phospho-Tau217 levels with tau positron emission tomography in early Alzheimer disease. JAMA Neurol 2021; 78: 149–156. 33165506 10.1001/jamaneurol.2020.4201 PMC7653537

[20] Ashton NJ Brum WS Di Molfetta G , et al. Diagnostic accuracy of a plasma phosphorylated Tau 217 immunoassay for Alzheimer disease pathology. JAMA Neurol 2024; 81: 255–263. 38252443 10.1001/jamaneurol.2023.5319 PMC10804282

[21] Mattsson-Carlgren N Janelidze S Palmqvist S , et al. Longitudinal plasma p-tau217 is increased in early stages of Alzheimer's disease. Brain 2020; 143: 3234–3241. 33068398 10.1093/brain/awaa286 PMC7719022

[22] Du L Langhough RE Wilson RE , et al. Longitudinal plasma phosphorylated-tau217 and other related biomarkers in a non-demented Alzheimer's risk-enhanced sample. Alzheimers Dement 2024; 20: 6183–6204. 38970274 10.1002/alz.14100 PMC11497664

[23] Lantero Rodriguez J Karikari TK Suarez-Calvet M , et al. Plasma p-tau181 accurately predicts Alzheimer's disease pathology at least 8 years prior to post-mortem and improves the clinical characterisation of cognitive decline. Acta Neuropathol 2020; 140: 267–278. 32720099 10.1007/s00401-020-02195-x PMC7423866

[24] Moscoso A Grothe MJ Ashton NJ , et al. Longitudinal associations of blood phosphorylated Tau181 and neurofilament light chain with neurodegeneration in Alzheimer disease. JAMA Neurol 2021; 78: 396–406. 33427873 10.1001/jamaneurol.2020.4986 PMC7802009

[25] Moscoso A Grothe MJ Ashton NJ , et al. Time course of phosphorylated-tau181 in blood across the Alzheimer's disease spectrum. Brain 2021; 144: 325–339. 33257949 10.1093/brain/awaa399 PMC7880671

[26] Hansson O Cullen N Zetterberg H , et al. Plasma phosphorylated tau181 and neurodegeneration in Alzheimer's disease. Ann Clin Transl Neurol 2021; 8: 259–265. 33249783 10.1002/acn3.51253 PMC7818141

[27] Chen SD Huang YY Shen XN , et al. Longitudinal plasma phosphorylated tau 181 tracks disease progression in Alzheimer's disease. Transl Psychiatry 2021; 11: 356. 34120152 10.1038/s41398-021-01476-7 PMC8197760

[28] Mielke MM Dage JL Frank RD , et al. Performance of plasma phosphorylated tau 181 and 217 in the community. Nat Med 2022; 28: 1398–1405. 35618838 10.1038/s41591-022-01822-2 PMC9329262

[29] Antonioni A Raho EM Di Lorenzo F . Is blood pTau a reliable indicator of the CSF status? A narrative review. Neurol Sci 2024; 45: 2471–2487. 38129590 10.1007/s10072-023-07258-x

[30] Kilander L Berglund L Boberg M , et al. Education, lifestyle factors and mortality from cardiovascular disease and cancer. A 25-year follow-up of Swedish 50-year-old men. Int J Epidemiol 2001; 30: 1119–1126. 11689532 10.1093/ije/30.5.1119

[31] Folstein MF Folstein SE McHugh PR . Mini-mental state”. A practical method for grading the cognitive state of patients for the clinician. J Psychiatr Res 1975; 12: 189–198. 1202204 10.1016/0022-3956(75)90026-6

[32] Franzon K Zethelius B Cederholm T , et al. Modifiable midlife risk factors, independent aging, and survival in older men: report on long-term follow-up of the Uppsala longitudinal study of adult men cohort. J Am Geriatr Soc 2015; 63: 877–885. 25919442 10.1111/jgs.13352

[33] Franzon K Zethelius B Cederholm T , et al. The impact of muscle function, muscle mass and sarcopenia on independent ageing in very old Swedish men. BMC Geriatr 2019; 19: 153. 31142271 10.1186/s12877-019-1142-y PMC6542054

[34] Velickaite V Giedraitis V Strom K , et al. Cognitive function in very old men does not correlate to biomarkers of Alzheimer's disease. BMC Geriatr 2017; 17: 208. 28886705 10.1186/s12877-017-0601-6 PMC5591537

[35] Koedam EL Lehmann M van der Flier WM , et al. Visual assessment of posterior atrophy development of a MRI rating scale. Eur Radiol 2011; 21: 2618–2625. 21805370 10.1007/s00330-011-2205-4 PMC3217148

[36] Pasquier F Leys D Weerts JG , et al. Inter- and intraobserver reproducibility of cerebral atrophy assessment on MRI scans with hemispheric infarcts. Eur Neurol 1996; 36: 268–272. 8864706 10.1159/000117270

[37] Scheltens P Leys D Barkhof F , et al. Atrophy of medial temporal lobes on MRI in “probable” Alzheimer's disease and normal ageing: diagnostic value and neuropsychological correlates. J Neurol Neurosurg Psychiatry 1992; 55: 967–972. 1431963 10.1136/jnnp.55.10.967 PMC1015202

[38] Ronnemaa E Zethelius B Lannfelt L , et al. Vascular risk factors and dementia: 40-year follow-up of a population-based cohort. Dement Geriatr Cogn Disord 2011; 31: 460–466. 21791923 10.1159/000330020

[39] American Psychiatric Association . Diagnostic and Statistical Manual of Mental Disorders, Fourth Edition. Washington, DC: American Psychiatric Association, 1994.

[40] McKhann G Drachman D Folstein M , et al. Clinical diagnosis of Alzheimer's disease: report of the NINCDS-ADRDA work group under the auspices of department of health and human services task force on Alzheimer's disease. Neurology 1984; 34: 939–944. 6610841 10.1212/wnl.34.7.939

[41] Syvanen AC Sajantila A Lukka M . Identification of individuals by analysis of biallelic DNA markers, using PCR and solid-phase minisequencing. Am J Hum Genet 1993; 52: 46–59. 8434605 PMC1682118

[42] Charlson ME Pompei P Ales KL , et al. A new method of classifying prognostic comorbidity in longitudinal studies: development and validation. J Chronic Dis 1987; 40: 373–383. 3558716 10.1016/0021-9681(87)90171-8

[43] Quan H Sundararajan V Halfon P , et al. Coding algorithms for defining comorbidities in ICD-9-CM and ICD-10 administrative data. Med Care 2005; 43: 1130–1139. 16224307 10.1097/01.mlr.0000182534.19832.83

[44] Milà-Alomà M Ashton NJ Shekari M , et al. Plasma p-tau231 and p-tau217 as state markers of amyloid-β pathology in preclinical Alzheimer's disease. Nat Med 2022; 28: 1797–1801. 35953717 10.1038/s41591-022-01925-w PMC9499867

[45] Ossenkoppele R Reimand J Smith R , et al. Tau PET correlates with different Alzheimer's disease-related features compared to CSF and plasma p-tau biomarkers. EMBO Mol Med 2021; 13: e14398. 10.15252/emmm.202114398 PMC8350902 34254442

[46] Palmqvist S Insel PS Stomrud E , et al. Cerebrospinal fluid and plasma biomarker trajectories with increasing amyloid deposition in Alzheimer's disease. EMBO Mol Med 2019; 11: e11170. 10.15252/emmm.201911170 PMC6895602 31709776

[47] Therriault J Servaes S Tissot C , et al. Equivalence of plasma p-tau217 with cerebrospinal fluid in the diagnosis of Alzheimer's disease. Alzheimers Dement 2023; 19: 4967–4977. 37078495 10.1002/alz.13026 PMC10587362

[48] Tang Y Whitman GT Lopez I , et al. Brain volume changes on longitudinal magnetic resonance imaging in normal older people. J Neuroimaging 2001; 11: 393–400. 11677879 10.1111/j.1552-6569.2001.tb00068.x

[49] Zhang Y Qiu C Lindberg O , et al. Acceleration of hippocampal atrophy in a non-demented elderly population: the SNAC-K study. Int Psychogeriatr 2010; 22: 14–25. 19958567 10.1017/S1041610209991396

[50] Nelson PT Dickson DW Trojanowski JQ , et al. Limbic-predominant age-related TDP-43 encephalopathy (LATE): consensus working group report. Brain 2019; 142: 1503–1527. 31039256 10.1093/brain/awz099 PMC6536849

[51] Nichols E Merrick R Hay SI , et al. The prevalence, correlation, and co-occurrence of neuropathology in old age: harmonisation of 12 measures across six community-based autopsy studies of dementia. Lancet Healthy Longev 2023; 4: e115–e125. 10.1016/S2666-7568(23)00019-3 PMC9977689 36870337

[52] Jansen WJ Janssen O Tijms BM , et al. Prevalence estimates of amyloid abnormality across the Alzheimer disease clinical spectrum. JAMA Neurol 2022; 79: 228–243. 35099509 10.1001/jamaneurol.2021.5216 PMC12138908

[53] Roberts RO Aakre JA Kremers WK , et al. Prevalence and outcomes of amyloid positivity among persons without dementia in a longitudinal, population-based setting. JAMA Neurol 2018; 75: 970–979. 29710225 10.1001/jamaneurol.2018.0629 PMC6142936

[54] Mielke MM Fowler NR . Alzheimer disease blood biomarkers: considerations for population-level use. Nat Rev Neurol 2024; 20: 495–504. 38862788 10.1038/s41582-024-00989-1 PMC11347965

[55] Alzheimer's Association . 2021 Alzheimer's disease facts and figures. Alzheimers Dement 2021; 17: 327–406. 33756057 10.1002/alz.12328

[56] Palmqvist S Tideman P Mattsson-Carlgren N , et al. Blood biomarkers to detect Alzheimer disease in primary care and secondary care. JAMA 2024; 332: 1245–1257. 39068545 10.1001/jama.2024.13855 PMC11284636

[57] Brum WS Cullen NC Janelidze S , et al. A two-step workflow based on plasma p-tau217 to screen for amyloid beta positivity with further confirmatory testing only in uncertain cases. Nat Aging 2023; 3: 1079–1090. 37653254 10.1038/s43587-023-00471-5 PMC10501903

[58] Savva GM Wharton SB Ince PG , et al. Age, neuropathology, and dementia. N Engl J Med 2009; 360: 2302–2309. 19474427 10.1056/NEJMoa0806142

[59] Hazan J Liu KY Costello H , et al. Challenges in a biological definition of Alzheimer disease. Neurology 2024; 103: e209884. 10.1212/WNL.0000000000209884 PMC11449396 39353147

