# Supplementary material for: Plasma p-tau217 correlates strongly with cerebrospinal fluid Aβ42 and increases over a ten-year period in amyloid-positive, non-demented very old men
Source: J Alzheimers Dis. 2025 Oct 29;108(4):1778–89. doi: 10.1177/13872877251390387 (PMC12664933; doi:10.1177/13872877251390387)
Supplement: sj-docx-1-alz-10.1177_13872877251390387 - Supplemental material for Plasma p-tau217 correlates strongly with cerebrospinal fluid Aβ42 and increases over a ten-year period in amyloid-positive, non-demented very old men [file sj-docx-1-alz-10.1177_13872877251390387.docx]

**Supplemental Material**

**Plasma p-tau_217_ correlates strongly with cerebrospinal fluid Aβ_42_ and increases over a ten-year period in amyloid-positive, non-demented very old men**


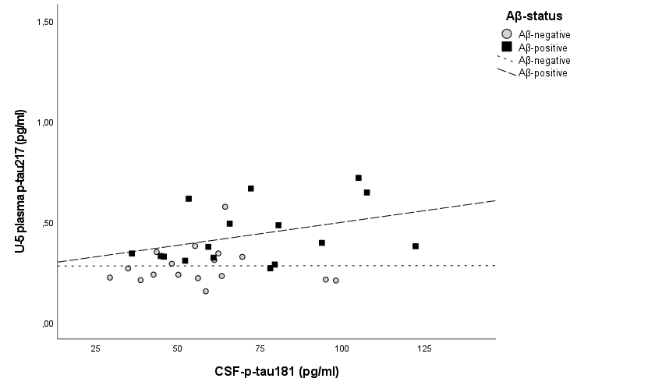

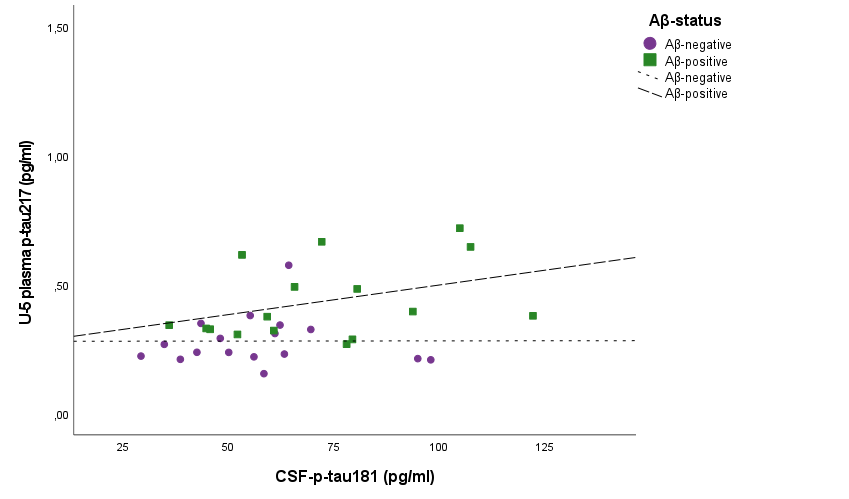


**Supplemental Figure 1a.** Scatter plots of CSF p-tau_181_ and plasma p-tau_217_ seven years before CSF sampling (U-5).


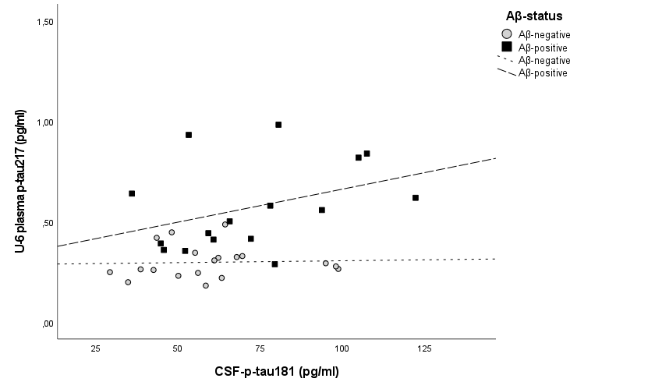

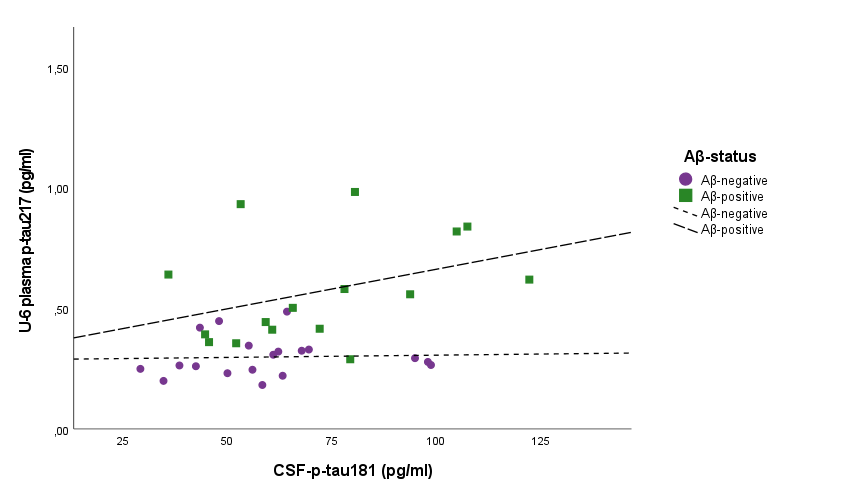


**Supplemental Figure 1b.** Scatter plots of CSF p-tau_181_ and plasma p-tau_217_ two years before CSF sampling (U-6, baseline).


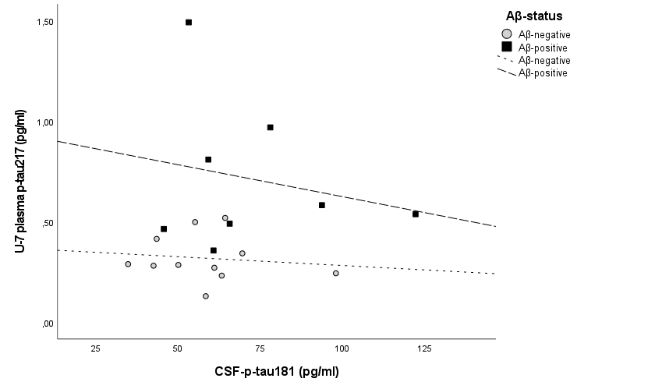

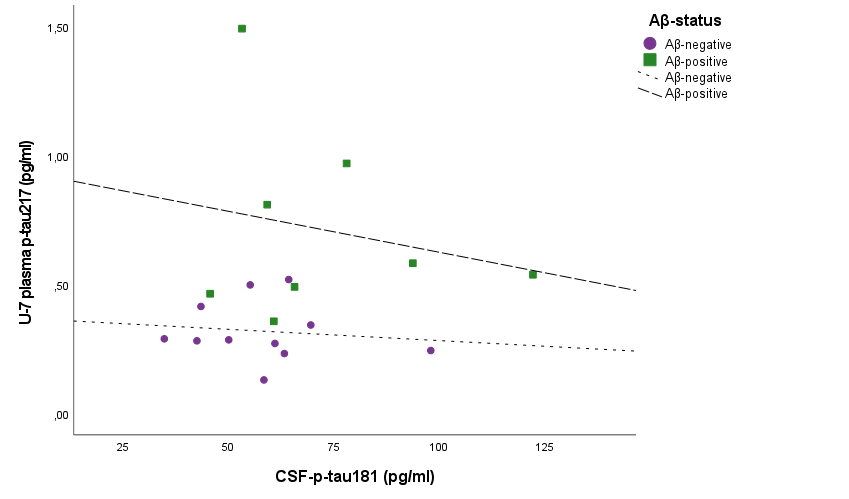


**Supplemental Figure 1c**. Scatter plots of CSF p-tau_181_ and plasma p-tau_217_ three years after CSF sampling (U-7).
